# A Rare Incidence of Sweating Sickness-Like Symptoms in a Crossbred Holstein Friesian Cow in Chattogram, Bangladesh

**DOI:** 10.1155/2023/6470133

**Published:** 2023-06-06

**Authors:** Omar Faruq, Eti R. Sarkar, Suchandan Sikder

**Affiliations:** ^1^Department of Medicine & Surgery, Chattogram Veterinary and Animal Sciences University, Khulshi, Chattogram, Bangladesh 4225; ^2^Department of Biochemistry and Biotechnology, Faculty of Basic Medical and Pharmaceutical Sciences, University of Science and Technology Chittagong, Chattogram 4225, Bangladesh

## Abstract

In this report, an incidence of sweating sickness-like symptoms in a crossbred Holstein Friesian cow was diagnosed. The cow was suffering from vaporization of the skin, dehydration, wet hair coat, and matting of hair due to excessive sweating. There were several ticks, flies, and mosquitoes in tail switch and other parts of the body. Blood and urine parameters were tested. We treated the patient successfully with ivermectin as ectoparasite control, ceftiofur sodium antibiotic to treat bacterial infections, ketoprofen as analgesics and antipyretics, chlorpheniramine maleate as H_2_-blocker, and trichlorfon and povidone-iodine skin spray to prevent fly invasion and prevent opportunistic bacterial infection, respectively. Acyclovir and oil of turpentine were suggested to be sprayed on the floor and wall of the shed for viral and ectoparasitic control. Our treatment regime successfully recovered the cow with no recurrence.

## 1. Introduction

Sweating sickness is a tick-borne toxicosis characterized by fever, moist dermatitis, and hyperemia of the skin and visible mucous membranes. It affects cattle of all ages although young calves are more susceptible [1]. However, experimental infections in sheep, goats, pigs, and dogs were successful [2]. The disease is caused by an epitheliotropic toxin (27-33 kDA) produced by certain strains of the female tick *Hyalomma truncatum* in their salivary glands. The toxin develops in the tick, not in the vertebrate host. The disease is prevalent in hot-humid climates of Eastern, Central, and Southern Africa, Sri Lanka, and Southern India. Incubation period and severity of the disease are dependent on the length of exposure and dose of toxin. If the exposure is >5 days, severe clinical signs and death may result sometimes. In subacute cases, the course is more prolonged, and recovery may occur. Mortality in affected calves is 30-70% under natural conditions [3]. Morbidity in endemic areas is about 10%. The severity of infection is influenced by the number of ticks and by the length of time they remain with the host. Recovered animals develop sustained immunity that may last ≥4 years.

Incubation period of sweating sickness is about 11 days following exposure to ticks. Sudden onsets of hyperthermia, anorexia, listlessness, ocular and nasal discharge, hyperemic mucous membranes, salivation, oral mucosal necrosis, and hyperesthesia are the predominant clinical findings [4]. If no care is taken, the affected animal may develop blindness due to sticking together of eyelids. Hot skin that rapidly worsens to moist dermatitis and skin becomes extremely sensitive and emits a sour and foul odor [1]. Most skin lesions appear at the base of the ears, axillae, groin, and perineum and extend over the entire body. Later, the hair and epidermis can be readily pulled off, exposing red and raw wounds. The tips of the ears and the tail may slough. The skin becomes hard and cracked and predisposed to secondary infection or screwworm infestation. Affected animals become difficult to handle, and movements become painful. The disease course is rapid, and death may happen within a few days.

In addition to the dermal lesions, the animal may also suffer from cachexia, dehydration, diphtheroid stomatitis, pharyngitis, laryngitis, esophagitis, vaginitis or posthitis, edema and hyperemia of the lungs, atrophic spleen, and hepatic, nephrotic, and meningeal congestion. Experimentally infected adult cattle develop moist dermatitis accompanied by a marked leukopenia [4]. Desquamation of the superficial layers of the mucous membranes of the upper respiratory, digestive tracts, and external genitalia. Identification of the toxin in affected cattle is quite difficult [3]. Presence of a tick vector and significant clinical findings are the basis to diagnose the disease. Effective treatment that typically needs hyperimmune serum, however, has been associated with problems of availability of health and recovered donors, possible serum contamination, and i.v. administration of large volume of serum [2, 5]. Alternative treatment requires removal of ticks, symptomatic treatment, and good nursing. Nonnephrotoxic antibiotics and anti-inflammatory drugs are recommended to prevent secondary bacterial infections. Control of vector is the only effective preventive measure. Regular acaricidal medication is suggested.

We aimed to write this report as sweating sickness-like symptoms has never been reported in cattle located in Chattogram and other areas of Bangladesh. Most owners and veterinarians are unaware of the disease. Reporting the current case will notify the farmers and veterinarians about the disease incidence to take steps to prevent the disease. Our current therapeutic guidelines will help to treat the animal successfully.

## 2. Case Presentation

A Holstein Friesian crossbred cow was suffering from bloody sweating at the neck and shoulder region and was associated with itching and inappetence. The cow was 6 years old and had approximately 350 kg body weight. The patient was from Baluchara region of Hathazari Upazila, Chattogram, Bangladesh, and referred to the Department of Medicine and Surgery, Chattogram Veterinary and Animal Sciences University (CVASU). The owner complained that the cow was initially suffering from a sudden onset of smoky evaporation from its neck and shoulder region and was associated with sweating tinged with blood. The signs appeared mostly during the evening and nighttime and disappear during daytime. The cow showed signs of anxiety and inappetence during sweating. There was no history of vaccination; however, anthelmintic medications performed on an irregular basis. The animal was treated with 3^rd^ generation cephalosporin ceftriaxone at 30 mg/kg body weight along with antihistamines and nonsteroidal anti-inflammatory drugs (NSAID) for 5 days but was not successful.

### 2.1. Investigations

**Figure 1:**
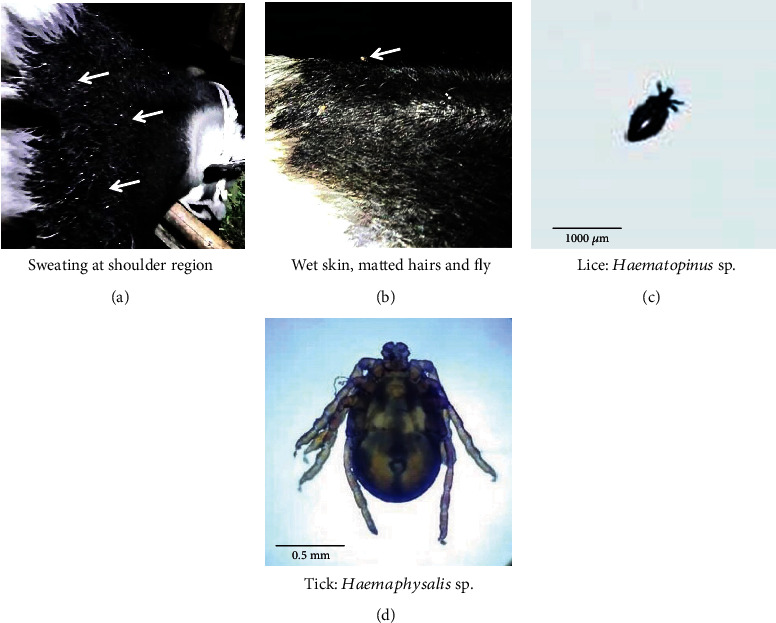
Sweating sickness-like symptoms in a cow. (a) Area of excessive sweating (indicated by arrows) represented by glistening on exposure to light; (b) wet skin, matting of hair, and presence of fly (arrow). (c) *Haematopinus* sp. lice and (d) *Haemaphysalis* sp. ticks were identified by their morphology and microscopic examination.

We attended the patient at evening time and observed hyperemic skin and profuse bloody sweating at the neck and shoulder region that made the skin wet (Figures 1(a) and 1(b)). The hair coat was rough and stray, and there was distinct evaporation. The animal was found weak and had pale mucous membrane, moderate dehydration, and normal superficial lymph nodes. The rectal temperature was 101.6°F, respiration rate 40/minute with no abnormal sounds, pulse rate 84/minute, and rumen motility was 4 per 2 minutes. The defecation and urinations were found normal. Upon clinical examination, an enormous number of *Haemaphysalis* sp. ticks, *Haematopinus* sp. lice, flies, and mosquitoes were found on the entire body surface with majority of them in the tail switch (Figures 1(c) and 1(d)). Ticks and lice were identified by morphological examination using light microscope by experts from the Department of Pathology and Parasitology, CVASU. Based on the owner's complaints, detailed clinical history, clinical signs, and examination findings, we presumptively diagnosed that the cow might have been suffering from sweating sickness.

After physical and clinical examination, blood, feces, and urine samples were collected for further investigation. Ten milliliters of blood was collected from the jugular vein with an 18G needle, and half of the volume was transferred to a clot activating tube vials to separate serum and the rest to a purple capped vacutainer having K2-EDTA in it. Five hundred milliliters of midstream urine sample was collected in a sterile container. Using sterile hand gloves, a 100 g fecal sample was collected directly from the rectum. The samples were transported to the physiology, parasitology, and clinical laboratories of CVASU using an icebox.

The whole blood sample was tested using a blood analyzer for hemoglobin (Hb) level, erythrocyte sedimentation rate (ESR), total erythrocyte count (TEC), total leukocyte count (TLC), mean corpuscular volume (MCV), red cell distribution width (RDW), and differential leukocyte count (DLC) [6]. Using a serum analyzer, the serum sample was analyzed to measure calcium (Ca), phosphorus (P), magnesium (Mg), glucose, total protein, and hepatic enzymes aspartate aminotransferase (AST) and alanine aminotransferase (ALT) following protocols previously described [7]. To detect blood protozoa, the Giemsa staining of thin, thick, and wet smear of blood was performed and examined under light microscope [8]. Coproscopy was performed following standard procedures of direct smear, floatation, and sedimentation to detect gastrointestinal worm load in the affected cow [9]. Using a urine analyzer, urine pH, glucose, protein, ketones, creatinine, etc., were measured [10]. It was observed that the animal was suffering from neutrophilia (Table 1). The parasitological examination of blood and feces was negative.

Serum analysis of the cow showed lower levels of calcium and total protein (Table 2). Surprisingly, there was an elevated level of AST and a lower level of ALT.

Urinalysis of the cow revealed that there was increased creatinine level and acidity of urine when the disease was in clinical form. However, the creatinine level and pH came back to normal range following treatment (Table 3).

### 2.2. Differential Diagnosis

As the skin is the largest organ of the body and barrier to the surroundings, it is affected by a wide range of infectious and noninfectious agents. We carefully assessed the differential diagnosis of the case with viral, bacterial, parasitic, allergic, and physical- and chemical-induced dermatitis [11]. We excluded lumpy skin disease as typical signs are acute onset of papules and nodules with rapid progressing to necrosis, sloughing off the skin, ulcer, and scar formation that were not found in the current case. We excluded foot-and-mouth disease and cow pox as these diseases have significant clinical lesions with vesicles and bullae formation, painful erosions, and ulcers in the mouth and interdigital space, udder, and teats [12]. We were unable to perform a definitive diagnosis for viral, bacterial, or toxicological etiology due to budget limitations. Bacterial infections might be present as a secondary infection. Moreover, dermatitis by physical, chemical, and sunlight exposure was excluded by carefully taken clinical history and absence of pasture grazing. Congenital and inherited epitheliogenesis imperfecta and edema disease were excluded as these diseases occur in calves at an early age [12]. Atopic dermatitis and urticaria were ruled out by the absence of urticarial plaque and bullae formation. The clinical case is more resemblance to sweating sickness of cattle; however, we keep out this diagnosis because of the absence of *Hyalomma truncatum* tick and unable to identify the toxin.

### 2.3. Treatment

The animal was treated with ivermectin at 0.2 mg/kg body weight (BW) subcutaneously (s.c.) with a 3-day interval for 4 times, antibiotic ceftiofur sodium at 2.85 mg/kg BW intramuscularly (i.m.) once daily for 5 days to prevent secondary bacterial invasion, and NSAID ketoprofen at 3 mg/kg BW i.m. once daily for 4 days [11] as specific antitoxin or hyperimmune serum was not available. Histamin-2 blocker chlorpheniramine maleate at 0.5 mg/kg BW i.m. once daily for 5 days was injected to prevent unexpected immune reaction to ceftiofur [13]. Trichlorfon was suggested to pour on by avoiding the lesions to treat ectoparasites [14]. Povidone-iodine spray was suggested on the wet skin once daily for 10 days to prevent opportunistic bacterial infections [15]. Acyclovir and oil of turpentine were suggested to be sprinkled on the shed to prevent viruses, fly, and ticks [16, 17]. The animal was treated with Ringer's acetate 1000 mL intravenous along with amino acid and iron preparations once daily for 3 days as hypoproteinemia was diagnosed [18]. All samples were collected and tested a second time two weeks after completion of the treatment provided.

### 2.4. Outcome and Follow-Up

We monitored the animal during and after treatment. Immediately after the start of treatment, the clinical signs started to disappear. The sweating and evaporation of the cow became diminished; there was absence of fly, tick, or lice at the end of treatment. Most of the blood, serum, and urine parameters became normal after treatment was completed (Tables 1–3). We maintained a regular mobile phone contact with the animal owner to monitor recurrence of the clinical signs but was negative after one month of treatment.

## 3. Discussion

Sweating sickness is a rare tick-borne illness in cattle and possibly other ruminants that is reported occasionally in southern India and African countries. In Bangladesh, there is no reported case until today. Major clinical signs identified from the current case were moist dermatitis on thoracic region with evaporation of the skin, rough hair coat, matted hair due to exudation, and presence of numerous lice and mosquitoes all over the body. Other symptoms recorded were dehydration, fever, anorexia, and pale mucous membrane. The clinical signs and findings are significantly similar to the signs described in cases with sweating sickness in cattle [1, 4]. Although the current case is similar to sweating sickness of cattle which is caused by the toxin produced by *Hyalomma* tick [3], we identified *Haemaphysalis* sp. of ticks from the cow. It would be ideal to identify the toxin produced by *Haemaphysalis* sp. ticks and causing similar disease in cattle. Other clinical signs typical to sweating sickness but were not identified in the current case are oculonasal discharge, blindness due to dehydration, hard and crack skin, hyperesthesia, and desquamation of the mucous membranes of upper gastrointestinal tract, respiratory tract, and genital tract [4]. Early intervention with proper treatment might have prevented the worsening of the current disease conditions.

Neutrophilia observed in the affected cow might be due to secondary bacterial infections [19], although Dolan and Newson [4] reported leukopenia and neutropenia in experimental sweating sickness in cattle. However, it would be interesting to identify any toxic effects on neutrophils or the presence of band cells. Proteinuria observed in the current scenario might be another cause of hypoproteinemia. Alternatively, toxemia might have contributed to the drop of serum albumin and/or globulins as an expected finding in the cases of sweating sickness [1]. However, we were unable to determine which component of serum protein was reduced. Furthermore, toxemia might have contributed to the development of nephrosis and subsequent proteinuria and creatinuria observed in the cow [10]; however, determination of serum creatinine level could provide more reliable information of kidney damage [20]. We also observed low mean corpuscular volume (MCV) which indicates microcytic anemia and might be due to toxemia or iron deficiency [21]. Cattle with lower MCV are susceptible to high parasitic infestation such as theileriosis [22]. We were unable to identify which one happened earlier than the other. However, after treatment, the blood values became within the normal range.

We observed hypocalcemia in the present case, and this might have contributed to the bloody sweating. Hypocalcemia generally occurs due to the high demand of calcium in the dairy cows [23], especially during lactation and late pregnancy [24]. Extracellular calcium is essential for bone formation, nerve impulse transmission, muscle contraction, blood clotting, and during metabolism. In the present case, hypocalcemia might not be related to sweating sickness, however might be due to milk yield and inappetence.

Glutamate dehydrogenase and gamma glutamyl transferase are mirror hepatic enzymes in ruminants to detect liver function [25, 26]. However, we were able to test aspartate aminotransferase (AST) and alanine aminotransferase (ALT), which are normally predominantly contained within liver cells and to a lesser degree in muscle cells. Upon hepatic injury, liver cells spill these enzymes into the blood, raising the AST and ALT enzyme blood levels and signaling liver disease [11]. We observed higher levels of AST in the cow that came back to normal following treatment. High AST levels might be due to the toxin having hepatic and/or muscle injury. Alternatively, the subcutaneous (s.c.) muscle of the affected skin area might have been affected. An ultrasonographic examination of the muscle could further clarify the involvement of s.c. muscle. No blood and intestinal parasites were observed in the current case that might be due to the previous treatment with anthelmintics.

We provided treatment with antibiotics to prevent secondary bacterial infection, antihistamines to mitigate adverse reactions of antibiotics, analgesics to reduce inflammation, Ringer's acetate to dilute the toxin and restore electrolytes and pH, and oil of turpentine and trichlorfon for ectoparasite control. Ceftiofur is an antibiotic commonly used in bacterial infections in cattle [12]. In addition to ceftiofur, we sprayed povidone iodine locally on the skin to limit opportunistic infection and facilitate wound healing [15]. Ketoprofen used in this case was recommended in the diseases of cattle where fever is present [12]. We treated the patient with H2-antagonist chlorpheniramine maleate to ensure zero risk of IgE-mediated type I hypersensitivity reactions against ceftiofur antibiotic [13]. Ectoparasitic nuisance of the cow was treated with therapeutic doses of trichlorfon, a parasiticide frequently used in ruminants [12]. Moreover, to minimize possible viral and fly prevalence, we suggested to spray acyclovir and oil of turpentine on the ground. These antiviral and fly repellents are recommended to prevent herpes virus infection and myiasis, respectively [16, 17]. Finally, to restore blood volume, we prescribed Ringer's acetate as this intravenous infusion is safe and frequently administered in cattle with dehydration [18]. Surprisingly, the cow recovered upon progression of treatment, indicating that the treatment was effective. We suggest that taking prompt action with our recommended treatment strategy could alleviate sweating sickness-like symptoms in cattle.

## Figures and Tables

**Table 1:** Blood parameters before and after treatment.

Blood parameters	Standard ranges	Before treatment	After treatment
Hb (g/dL)	8-15	7.7	9.3
ESR (mm/h)	0-3	0	0
TEC (million/cmm)	4-12	6.05	8
TLC (thousand/cmm)	5-10	8.7	7.5
Neutrophils (%)	15-33	55	35
Eosinophils (%)	0-20	5	3
Lymphocytes (%)	45-75	35	43
Monocytes (%)	0-8	5	3
Basophils (%)	0-2	0	0
Platelets (lakhs/cmm)	1-8	4.63	5.35
MCV (fL)	40-60	36.2	46.03
MCH (pg)	5.2-8	12.7	7.7
MCHC (g/dL)	31-38.5	35.2	36.3
RDW (%)	0-99.9	16.4	48.5
MPV (fL)	3.5-6.5	5.2	5.6

Hb: hemoglobin; ESR: erythrocyte sedimentation rate; TEC: total erythrocyte count; TLC: total leukocyte count; MCV: mean corpuscular volume; MCH: mean corpuscular hemoglobin; MCHC: mean corpuscular hemoglobin concentration; RDW: red cell distribution width; MPV: mean platelet volume.

**Table 2:** Serum analysis for minerals, glucose, total protein, and liver function test.

Serum parameters	Standard ranges	Before treatment	After treatment
Calcium (mg/dL)	8-11.4	6.9	8.21
Phosphorous (mg/dL)	5.5-8	6.85	5.22
Magnesium (mg/dL)	1.5-2.9	2.66	1.79
Glucose (mg/dL)	40-100	61.79	45.42
Total protein (g/dL)	6.7-7.5	5.5	4.7
AST (U/L)	11-40	96.9	8.82
ALT (U/L)	60-125	45.09	28.40

AST: aspartate aminotransferase; ALT: alanine aminotransferase.

**Table 3:** Urinalysis of cow before and after treatment.

Urine parameters	Standard ranges	Before treatment	After treatment
Specific gravity	1.02-1.05	1.05	1.03
Odor	Aromatic	Aromatic	Aromatic
Color	Straw	Straw	Straw
Urobilinogen (mg/dL)	Negative	2	2
Glucose (mg/dL)	Negative	Negative	Negative
Bilirubin	Negative	+ (low)	+ (low)
Ketone	Negative	Negative	Negative
pH	7-8.4	6	8
Protein	Negative	Negative	Negative
Creatinine (mg/dL)	45-75	100	10
Nitrate	Negative	Negative	Negative
Leukocyte (…/*μ*L)	Negative	>25	>25

## Data Availability

All data collected from the case are provided in the manuscript.
